# On the Diseases, Injuries and Malformations of the Rectum and Anus

**Published:** 1860-09

**Authors:** 


### BOOK AND PAMPHLET NOTICES.

On the Diseases, Injuries and Malformations of the Rectum and Anus. With Remarks on Habitual Constipation. By T. J. Ashton, M. D.

Blanchard and Lee have just issued a reprint of the third Edition of this valuable work, which made its appearance during the present year in London. This edition has been carefully re-written, with such additions as the author's extended experience permits him to make, to the present time. The present edition is finely illustrated, which gives it decided preference over the former ones.

These illustrations have been re-produced in the American edition, with the characterestic fidelity of the enterprising publishers.

The volume, containing nearly three hundred pages, describes in a concise and lucid manner, the various diseases to which this portion of the body is subject, and the most successful means of palliation and cure.

It treats of irritation and itching, of inflammation and excoriation, of excresences, contractions, fissure and neuralgia of the anus, devoting a chapter to each topic.

The causes, symptoms, complications and treatment of inflammation and ulceration of the rectum, comprise the two very valuable chapters next in order.

The subject of Hemorhoidal affections, with a citation of some thirty cases, with the treatment and result detailed, is of special interest and afford much valuable instruction upon this particular point.

Abscesses, fistula, polypi and strictures are briefly but ably treated of; while malignant diseases of the rectum, injuries, malformation and the effects of foreign bodies, are amply noticed.

The concluding chapter upon Habitual Constipation is worthy of particular attention, and if our space permitted, we should be inclined to present it entire for the benefit of our readers. The author remarks:—" Habitual Constipation is one of the most prevalent and troublesome functional disorders to which mankind is subject. Its sympathetic effects extend to every organ of the body, and often occasion great distress and anxiety to the sufferers, leading them to apprehend the existence of the most serious organic disease. Neither can it be doubted that many of the pathological changes in structure of the viscera of the head, chest and abdomen, have their origin in functional derangement, induced either sympathetically by constipation and consequent derangement of the assimulative organs, or by retention of excrementitious matter.

" Of the sympathetic effects upon the brain and nervous system thereby induced, we have evidence during infancy and youth, in convulsive fits, chorea and other nervous affections, and in adults in the giddiness, drowsiness, headache, pains extending to various parts of the body, and that distressing mental depression denominated hypochondroasis, which not unfrequently terminates in permanent perversion of intellect or even in a more distressing manner. The sympathetic effects upon the lungs and heart are indicated by cough and palpitation. The reaction on the stomach is marked by disordered appetite, vomiting, eructations and a sense of gnawing and sinking at the precordia. We have evidence of the kidneys being affected in their morbid secretions, as marked by the various deposits we find in the urine.

"The exhalent functions of the lungs and skin also become deranged, as indicated by the fœtor of the breath and perspiration; and many of the distressing and unsightly diseases of the skin, have their origin in constipation and morbid accumulations in the bowels.

"Nor do the genito-urinary organs escape; thus urethral, vaginal and uterine discharges and irritability of the bladder are frequently induced.

"The countenance of those who are the subjects of habitual constipation is dull and heavy—the eyes lack their lustre, and the tongue is observed to be deeply notched transversely."

He proceeds to remark upon the most common causes of constipation, depending upon torpor of the color, and the means of avoiding that condition. The *subjects* of this affection are those whose powers of life are naturally low—in the earlier period of life, more frequently delicate females; but as age advances and the organic functions become enfeebled, we find it prevailing in either sex.

The most frequent accidental *causes* are sedentary habit, and a very common practice of *not attending to the first calls of nature to evacuate the bowels.*

The loss of contractility of the intestines from enormous and frequently unnoticed distention of the colon, brings as its sequence a vast train of evils.

In the treatment of habitual constipation, the author adds, "the object to be obtained is the removal of the cause; to procure fæcal evacuations by the mildest and least irritating means adequate to the purpose; to restore the lost tone, and to prevent the recurrence of the torpid condition of the bowels."

The appropriate and the *injurious* agencies resorted to for the accomplishment of these purposes, are pointed out, and his judicious advice with reference to the *habits of attention* to the *state of the bowels*, with reference to *food exercise*, *occupation*, &c., will profit the practitioner and the patient. We deem it the best work of the kind which has yet been presented to the medical profession. J. H. H.

